# Ethnic and national identification of minority youth in China: The mediating role of intergroup orientation

**DOI:** 10.1371/journal.pone.0305169

**Published:** 2024-06-13

**Authors:** Ning Luo, Lei Wang, Tao Guan

**Affiliations:** 1 Department of Cultural and Creative Arts, The Education University of Hong Kong, Hong Kong SAR, China; 2 Department of Special Needs Education, University of Oslo, Blindern, Norway; 3 College of Music and Dance, Guangzhou University, Guangzhou, China; Universita Telematica Pegaso, ITALY

## Abstract

This study examined the mediating role of intergroup orientation in the relationship between ethnic and national identification. Our participants were 1320 Yi minority youths from a secondary school located in the Yi ethnic autonomous prefecture of southwest China. The participants completed three self-report questionnaires measuring ethnic and national identification, and intergroup orientation, respectively. Structural equation modeling was employed to determine the relationships between ethnic and national identification and intergroup orientation, and to explore the mechanism underlying the association between ethnic and national identification. The results showed that Yi minority youths with a stronger sense of ethnic identity had a stronger sense of national identity. The results further indicated that stronger ethnic identity led to a more positive intergroup orientation, which in turn predicted a stronger national identity. Our findings may facilitate the cultivation of positive attitudes between national subgroups in multiethnic countries and help ethnic minority youth develop a stronger awareness of national identity while retaining their ethnic identity.

## Introduction

In multiethnic countries, the interplay between ethnic identity, national identity, and attitudes between national subgroups (i.e., intergroup orientation) poses important implications for cultural diversity and social cohesion [1]. Ethnic identity denotes an individual’s self-identification derived from belonging to an ethnic group [2, 3], while national identity derives from the individual’s knowledge, value system, behavioral patterns, and emotions that they attach to their nation [4]. Research has shown that intergroup orientation may shape minority youth’s ethnic and national identification development [5], and thus contribute to the different ways in which they acculturate [6]. According to Berry [7], the assimilation approach requires minority youth to adopt the majority culture and discard their ethnic cultures, which may lead to conflict between their ethnic and national identities. In contrast, the integration approach encourages minority youth to embrace the mainstream culture while retaining their ethnic cultures, which may foster a nonconflicting association between their ethnic and national identities [7].

Existing acculturation theories are limited by their focus on the contexts of migration [8] and areas with intense ethnic conflicts [9, 10]. In the above contexts, minority youth struggle with combining their ethnic identities with the national identity [6], and negative intergroup orientations variously influence their identification processes [10]. Few studies have examined minority youth who have grown up in their indigenous areas where the ethnic cultures are encouraged locally as the mainstream. In this study, the Yi group is a long-standing ethnic group indigenous to Yi regions in China, with an established culture [11, 12]. The development of the ethnic and national identities and the intergroup orientations of Yi minority youth may vary according to the particular sociocultural context.

In the past two decades, the orthogonal model of ethnic and national identification has replaced the traditional unidimensional model [13, 14]. While the unidimensional model places the two types of identification at opposite ends of a continuum, the orthogonal model suggests that they are independent of each other, and that increasing identification in one dimension does not require decreasing identification in the other [7]. It suggests that minority youth can have a strong sense of ethnic identity while identifying with the larger society. However, the internal mechanism by which the orthogonal model operates is unclear. From a developmental perspective, Phinney [2] proposed that the attitudes of minority youth toward other national subgroups play a vital role in their forming of a superordinate national identity while retaining their ethnic identity. This suggestion underlines the effect of intergroup orientation on minority youth’s ethnic and national identification processes. Therefore, the present research aimed to fill a literature gap by examining the interplay between ethnic and national identification and the mediating role of intergroup orientation in Yi minority youth from a Yi ethnic region in China. Our findings shed light on theories of acculturation and on regional policies in multiethnic countries that aim to promote interethnic cohesion while maintaining cultural diversity.

### The sociocultural context of the Chinese Yi ethnic region in this study

China is a multiethnic country consisting of the Han ethnic majority and over 50 ethnic minorities (e.g., Yi, Mongols, Tibetans, Hmong, Bai, Buyi, Lisu, Qiang, and Tujia) who are distinguished by their geographic, linguistic, religious, and cultural characteristics [12]. Unlike in countries where ethnic and national identities may overlap significantly [15], these identities are two distinct yet interdependent concepts in China [16]. An ethnic group in China is defined by its ethnic characteristics and conformity to its culture, history, language, and religion [17]. In comparison, the Chinese national identity was formed around a shared political commitment against the threat of invasion in the late Qing dynasty in the early 20^th^ century [18]. Current nationalistic thought in China posits that the country’s diverse indigenous ethnic groups gradually developed to form the modern nation through their shared history of interaction.

Although ethnic minorities in China constitute about 6% of the total population, they form the majority groups in ethnic regions, which account for 64% of China’s total land territory [19]. For example, in the Yi ethnic region of the current study, which is located in southwest China, Yi people are the local majority and constitute 75% of the population [20]. Accordingly, there may be differences in ethnic and national identification between Yi- and Han-majority regional contexts. To balance ethnocultural inheritance with national integration in Yi ethnic regions, the Regional Ethnic Autonomy System [21] has been enacted to promote national identity not by enforcing assimilation but by encouraging multiculturalism [22]. By promoting the Chinese national identity as a superordinate identity, the REAS includes ethnic groups in a larger national group. It is implemented, especially through school education, to encourage Yi minority youth to fully explore their ethnicity inside and outside of school, thus enabling an inclusive regional environment where Yi culture is represented [11]. This strategy affords Yi youth increased opportunities to immerse themselves in their own ethnocultural practices, including language, festivals, and customs. Notably, celebrations like the Yi New Year and the Torch Festival are recognized and celebrated as mainstream holidays within the local community [23]. However, this example occurs in a region with a relatively positive discourse on combining ethnic and national identities, which fosters an environment where cultural practices and identities can coexist and be celebrated. Thus, Yi minority youth growing up in Yi ethnic regions may undergo different ethnic and national identification processes than minorities living in Han-majority areas and thus hold different attitudes toward other national subgroups.

## Review of the literature and development of hypotheses

### Ethnic and national identification

Underpinned by acculturation theory [6], studies have investigated the relationship between the ethnic and national identification of ethnic minorities in pluralistic societies. The orthogonal model suggests that the process of acculturation comprises two dimensions: maintaining aspects of one’s ethnic heritage and adapting to the main culture of one’s plural society. On the basis of these two dimensions, Berry [7] proposed four acculturation patterns, namely (a) assimilation, (b) separation, (c) integration, and (d) marginalization. Assimilation refers to the willingness to adapt to the majority culture at the expense of losing one’s ethnic or racial heritage and identity. Separation is defined as the preference for retaining one’s cultural heritage at the expense of adapting to the mainstream culture. Integration is described as the willingness to fit into the mainstream culture as well as retain aspects of one’s cultural heritage and identity. Finally, marginalization is defined as the unwillingness to both retain one’s cultural heritage or identity and adapt to the mainstream culture.

The theory of bicultural identity orientations extends beyond the orthogonal model, offering a nuanced understanding of how individuals in multicultural societies relate to two or more ethnocultural groups [24]. This theory is crucial for exploring the dynamics of cultural identification and shedding light on the intricate ways individuals navigate their affiliations and identifications within multicultural contexts. Comănaru and colleagues [25] developed a comprehensive model of bicultural identity orientations that encompasses five distinct dimensions: hybridity, monoculturalism, alternation, complementarity, and conflict. Each dimension provides insights into different aspects of cultural identification: hybridity represents a blending of cultural identities; monoculturalism denotes identification with only one culture; alternation refers to the situational switching between cultural identities; complementarity conveys a sense of compatibility between cultural identities; and conflict indicates perceived incompatibility between them. Consistent with Berry et al. [6], Firat & Noels [26] discovered that the bicultural identity orientations of hybridity and complementarity served as two potential identity integration strategies advantageous to individuals’ psychological wellbeing.

Most studies have confirmed the assimilation pattern [27], indicating that ethnic minorities tend to strive for assimilation into the prevailing culture of their plural society. Furthermore, a survey of 5,366 minority youths from 13 countries revealed that the relationship between ethnic and national identities could be positive, negative, or absent [6]. In another study, Sinclair, Sidanius, and Levin [28] argued that the association between ethnic and national identification was positive for national majority groups but absent or negative for minority groups. The above findings suggest that ethnic and national identification may be subject to contextual dynamics, as well as group status [15].

Research has found that the way in which national identity is conceptualized can impact the association between the ethnic and national identities of minorities [29]. If the ideology of nationalism underpins the cultivation of national identity in such a way that the nation-building agenda depends on the majority group’s cultural origins, it is difficult for ethnic minorities to have nonconflicting ethnic and national identification [30]. For instance, Europeans often conceptualize their national identity as deriving from their ancestry in the country [31] and thus consider minorities who are immigrants without long family histories in the country as less representative of the nation. However, studies on ethnic and national identification have mostly been carried out in regions where the national majority groups constitute most of the population, as opposed to regions with a large ethnic minority presence. One exception is Rumbaut [32], who found that in Cuban American-majority Miami, Cuban American adolescents considered their ethnic identity to constitute Americanness. This finding implies that in regions with large ethnic minority populations, there may be a positive relation between ethnic and national identities, as the minority culture may form both the local mainstream culture and part of the national culture [33]. Given the sociocultural context of the Yi ethnic region in our study, where the Yi group constitutes the majority population and the Yi culture represents the local mainstream, we hypothesized that the ethnic identity of the region’s Yi minority youth is positively correlated with their national identity (Hypothesis 1).

### Intergroup orientation

Intergroup orientation refers to attitudes toward and interaction with ethnic groups other than one’s own [34]. Research has indicated that positive intergroup orientation is a key variable in ethnic and national identification processes [35], and has examined intergroup orientation as an outcome variable by focusing on how an individual’s ethnic and national identification influence their attitudes toward other national subgroups in different acculturation contexts [36–38]. For instance, Ellemers et al. [39] reported that a secure ethnic identity was characterized by a strong commitment to one’s ethnic group, and that this commitment was associated with lowered feelings of threat from other ethnic groups. Ashmore et al. [40] also found that minority youth who grew up in an inclusive context developed a secure ethnic identity, which contributed to their positive intergroup orientation. In the regional context of this study, the Yi group was represented in local culture, such that the Yi youth had opportunities to form a stable sense of ethnic self. Thus, we expected to find a positive association between ethnic identity and intergroup orientation among the Yi youth (Hypothesis 2).

Research on social categorization has suggested that if ethnic groups perceive themselves as belonging to a common superordinate category, their attitudes toward other national groups are positive, as they regard individuals from such groups as members of the same superordinate category [37]. For instance, Maloku et al. [10] found that in Kosovo, the degree to which Albanians and Serbs identified themselves with a common national identity predicted positive intergroup orientation. Likewise, Guan et al. [41] found that mainland Chinese individuals who highly identified with the superordinate Chinese identity demonstrated positive intergroup attitudes toward the Hong Kong Chinese subgroup.

These studies suggest that a more inclusive definition of national identity is associated with positive intergroup orientation. In China, the REAS encourages the concept of national identification as a supra-identity to define the Chinese nation, transcending ethnic, racial, and religious affiliations [17]. Therefore, we expected national identity to be positively correlated with intergroup orientation for the Yi youth in our study (Hypothesis 3).

### The mediating role of intergroup orientation

While acculturation theory [6] supports the model of orthogonality between ethnic identity and national identity, research elucidating the mechanism underlying this relation has been limited. In their comparison study of minority youth across five European countries, Fleischmann and Phalet [42] concluded that intergroup contexts play a vital role in minority youth’s identification pattern development. In particular, they suggested that positive intergroup orientation facilitates the validation of minorities’ dual identities. Hence, minorities hold conceptions of interethnic relations that are intertwined with those of ethnic and national identity [43].

Phinney [2] further posited that for minority youth growing up in inclusive cultural environments, a more robust sense of ethnic identity could lead to positive intergroup orientations because a stable sense of ethnic self is the basis for acceptance of and openness toward others. Positive intergroup orientations may then contribute to minority youth’s development of a superordinate identity conducive to social cohesion in a multiethnic society [44]. Given the above analysis and the inclusive environment endorsed by the REAS in the Yi ethnic region of our study, we hypothesized that the intergroup orientation of the region’s Yi minority youth plays a mediating role in the relationship between their ethnic identity and national identity (Hypothesis 4).

## The present study

Drawing on previous empirical studies, the model of orthogonality between ethnic and national identity [7], and the developmental perspective [2], this study aimed to investigate the association between ethnic and national identity, as well as the mediating effect of intergroup orientation on this relationship, in Yi minority youth from a Yi ethnic region in China. Our findings provide insights into how an acculturation strategy may be developed to allow minority youth to form a superordinate national identity while retaining their ethnic identity. This study explored the following research questions:

1. What is the relationship between ethnic identity, national identity, and intergroup orientation among Yi minority youth from a Yi ethnic region in China?
2. Does the Yi minority youth’s intergroup orientation mediate the relationship between their ethnic identity and national identity?

## Methodology

This research was a continuation of our project, “Identity Development of Adolescents in Ethnic Regions.” It maintained thematic coherence with our previous work by focusing on minority youth’s identity formation. However, this study distinctively focused on the Yi ethnic group, offering new insights into identity development in different ethnic contexts. Unlike our previous comparative analysis of multiple ethnic groups, this study provided a nuanced exploration of the Yi ethnic group’s unique context: a local majority. Another distinctive feature was this study’s emphasis on mediation, which allowed a deeper understanding of the relationships between ethnic and national identities and the uncovering of intermediary processes not explored in our previous work.

For this quantitative research, we gathered a large sample from our target population (i.e., Yi minority youth from the focal Yi ethnic region) to make generalizations about the focal factors (i.e., ethnic identity, national identity, and intergroup orientation) and identify patterns of interest. We determined the appropriate research paradigm for studying our participants’ identities to be post-positivism, which underlines how knowledge interpretation is affected by its specific sociocultural context [45]. Specifically, we employed the survey method as it can be used to gather data from a large sample efficiently, measure constructs that describe the nature of existing phenomena, and provide inferential information about certain correlations [46].

### Participants and sample size

The participants in the study were 1,320 Yi minority students (48.3% female) from a secondary school in the Yi ethnic autonomous prefecture of Sichuan province, China. The mean age of the students was 15.95 years (*SD* = 2.4). According to the students’ self-reports, 49% of their parents had not received formal schooling, 27% had received primary education, 23% had received secondary education, and 1% had received tertiary education. The family SES contexts for the parents of the participants were predominantly low and homogenous. Therefore, we excluded these variables, as they would have limited the predictive value for the relationships investigated in this study. Regarding sample size, Kline [47] suggested that at least 10 respondents per parameter is appropriate for a structural equation model. Wolf et al. [48] further suggested that a sample of 460 participants is appropriate for mediation testing. Given the nine parameters in this model (three regressions for the mediation model and six for the control variables), a sample size of 1,320 was appropriate for the mediation analysis in this study.

### Measures

#### Ethnic identity scale

Ethnic identity was measured using the Ethnic Identity Scale (EIS) developed by Qin [49]. The EIS was selected over other measures because it has been extensively validated for assessment of ethnic minorities in China [50]. The EIS contains 23 items that the participants rated on a 6-point Likert scale ranging from 1 = “strongly disagree” to 6 = “strongly agree,” with higher scores representing stronger ethnic identity. Example items are “I know some unique customs of the ethnic group I belong to,” and “It would be nice if I were not a member of the ethnic group I belong to (reverse coded)”. Cronbach’s alpha was.80. The score for this measure was the sum of the item scores.

#### Chinese national identity scale

The Chinese National Identity Scale (CNIS) of Qin and Zuo [51] was used to measure national identification because it has been validated in the Chinese population for diverse minority groups [52]. The CNIS is a self-report questionnaire containing 21 items. The respondents rated the items on a 6-point Likert scale ranging from 1 = “strongly disagree” to 6 = “strongly agree.” Higher scores indicated a stronger sense of Chinese national identity. A sample item of the CNIS is “I celebrate the traditional festivals of the Chinese nation.” Cronbach’s alpha was.83. The score for this measure was the sum of the item scores.

#### Intergroup orientation scale

Intergroup orientation was assessed using the Other-Group Orientation (OGO) scale [5]. The OGO scale is a 5-item scale that examines individuals’ attitudes toward other ethnic groups and their willingness to interact with members from these groups. Sample items are “I am willing to learn and work with people of other ethnic groups” and “I feel a sense of distance when I am with members of other ethnic groups (reverse coded).” The participants rated the items on a 6-point Likert scale ranging from 1 = “strongly disagree” to 6 = “strongly agree.” Higher scores indicated a more positive orientation toward other ethnic groups. Cronbach’s alpha was.67. The score for this measure was the sum of the item scores.

### Data collection procedure and ethical considerations

The study was approved by the human research ethics committee of the first author’s university. The data were collected in September 2019. The research team visited the secondary school site to meet with the school administrators and obtained parental consent. A research team member then visited the classes of Grade 9 and Grade 12 students to recruit participants. The researcher briefed the students on the purpose of the study and asked interested the students to remain in the classroom for filling questionnaires. Ninety-two per cent of the students decided to participate. They were instructed to answer freely and voluntarily and that their anonymity is guaranteed. An informed consent form for the participants was attached to the first page of the questionnaire. The participants took about 25 minutes to complete the questionnaires. After collection, the data was kept confidential and anonymous.

## Results

SPSS Version 25 [53] was used to explore the relationships between ethnic identity, national identity, and intergroup orientation. By testing the skewness and kurtosis of the data and using a single-sample Kolmogorov–Smirnov test, we found that our sample had a non-normal distribution (*p*s *<* .05). Therefore, we adopted a nonparametric statistical method (i.e., Spearman’s rank correlation) to calculate the correlations in our sample.

### Correlations between ethnic identity, intergroup orientation, and national identity

The Spearman’s rank correlation coefficients (Table 1) revealed that there was a moderately significant correlation between ethnic identity and national identity in the Yi students (*r*_*s*_ = .56, *p* < .001). Ethnic identity and intergroup orientation had a weak significant correlation (*r*_*s*_ = .25, *p* < .001), while national identity and intergroup orientation had a moderately significant correlation (*r*_*s*_ = .42, *p* < .001).

**Table 1:** Descriptive statistics and intercorrelations of the variables.

Variables	1	2	3
1. Ethnic identity	1.00		
2. National identity	.56***	1.00	
3. Intergroup orientation	.25***	.42***	1.00
Mean	108.57	105.32	24.82
Standard deviation	13.16	13.47	24.82
Cronbach’s *α*	.80	.83	.67

*** *p* < .001

### The mediation effect

A mediation model was tested to explore the mechanism underlying the relationship between ethnic identity and national identity. Specifically, ethnic identity was expected to contribute to national identity both directly and indirectly through the mediation of intergroup orientation. The model was estimated using the structural equation modeling function of the *lavaan* package [54] in R [55]. Specifically, bias-corrected 3000-sample bootstrapped standard errors were calculated for the parameters. The mediation effect was statistically significant if the 95% confidence interval for the parameter of the indirect pathway (ethnic identity → intergroup orientation → national identity) did not cover zero. Age and gender were controlled in the model.

**Fig 1:**
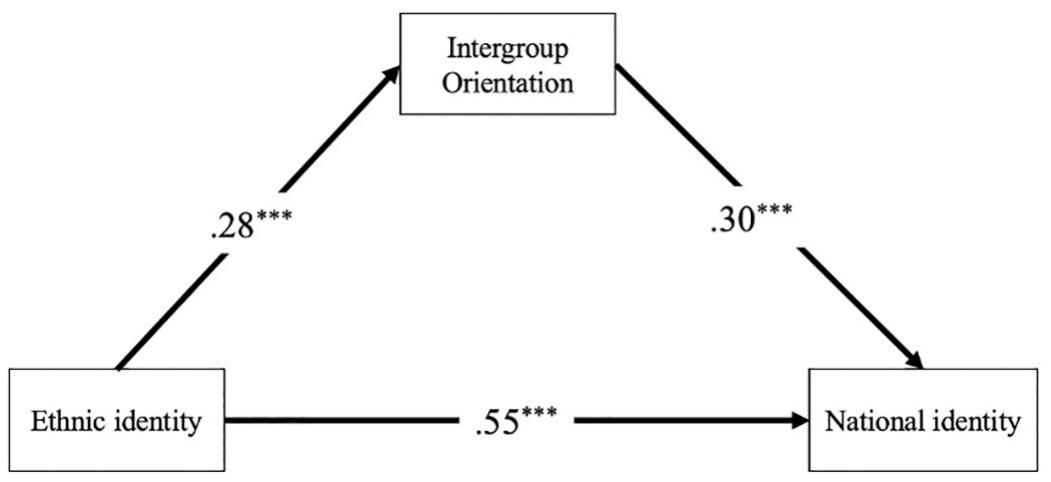
Mediation model with standardized coefficients included. Note: ***p < 0.001.

The results of the mediation model showed that intergroup orientation partially mediated the relation between ethnic identity and national identity. The standardized coefficients for the paths are shown in Fig 1. The direct path from ethnic identity to national identity (β = .55, *SE* = .03, *p* < .001), the path from ethnic identity to intergroup orientation (β = .28, *SE* = .01, *p* < .001), and the path from intergroup orientation to national identity (β = .30, *SE* = .07, *p* < .001) were all significant. The full indirect path (ethnic identity → intergroup orientation → national identity) was also significant (β = .08, *SE* = .01, *p* < .001, 95% confidence interval [.06,.12]). This indirect path corresponded to the unique contribution of ethnic identity to national identity mediated by intergroup orientation.

## Discussion

This study aimed to investigate the mediating role of intergroup orientation in the relationship between Yi minority students’ ethnic and national identities. As predicted, our findings showed that the relationship between ethnic identity and national identity was mediated by intergroup orientation. Our findings are important because they suggest that intergroup orientation can be made more positive by influencing ethnic identity, an outcome that may, in turn, strengthen national identity. A positive intergroup orientation may contribute to fostering minority youth’s sense of superordinate national identity while allowing them to retain a strong sense of ethnic identity.

First, following the model of orthogonality between ethnic and national identities [7], we hypothesized that ethnic identity is positively and significantly related to national identity for Yi minority youth living in the focal Yi ethnic region in China. The sample data confirmed this hypothesis. In agreement with this finding, several empirical studies have reported that youth from different ethnic groups may identify with a superordinate national identity while also retaining the distinctive features of their ethnic groups [36]. In the context of the current study, the REAS implemented is underpinned by an ethnically heterogeneous and collectivist culture, and promotes superordinate national identification without compromising individuals’ ethnic identity. This local policy acknowledges the cultural diversity in Yi ethnic regions and grants the Yi rights to decide their own affairs, thus allowing Yi minority youth to fully explore their ethnic identity in an inclusive environment [11]. Our finding suggests that minority youth growing up in regions with policies endorsing multiculturalism and good representation of ethnic cultures may maintain positive relationships between their ethnic and national identification.

Then, following Phinney’s developmental perspective [12], we conjectured that ethnic and national identification are positively related to intergroup orientation. The sample data also supported this hypothesis. The Yi minority youth participants with a stronger sense of ethnic and national identity reported more positive attitudes toward other national subgroup members. In contrast, research in other countries has found that increased ethnic identification is associated with negative intergroup attitudes as well as decreased national identification [56, 57]. However, the sociocultural contexts of these studies are different from that of our study’s Yi ethnic region, where the minority culture is promoted in school and an inclusive national identity is endorsed under the REAS. This environment enables Yi minority youth to better explore their ethnicity in the identity development process [11]. It might have increased the participants’ confidence in their ethnic identity, and thus provided them with the security to increase their understanding of other ethnic groups and intercultural thinking [58]. In line with the present findings, Gaertner and Dovidio [37] proposed that a nonconflicting relationship between ethnic and national identification may promote positive intergroup relations because a common superordinate category may help members of different groups regard themselves and members of outgroups as belonging to a more inclusive and superordinate group rather than two completely separate groups. This study underlines the relevance of strong ethnic and national identification to positive intergroup orientation in the Yi ethnic regional context, and suggests that local and national policies concerning ethnic and interethnic relations have been successful.

Finally, the results of this study showed that intergroup orientation partially mediated the relationship between the Yi minority youth participants’ ethnic and national identities. They indicated that intergroup orientation played a small but crucial role in maintaining a positive association between ethnic and national identification. Although there have been no empirical studies on the mediating role of intergroup orientation, related studies have demonstrated that attitudes toward other groups are associated with the construction of ethnic and national identities [59, 60]. By approaching superordinate national identification from an intergroup perspective and considering how subgroups can identify with a common identity without losing their separate identities, this study suggests that minority youth growing up in regions with policies endorsing multiculturalism and representation of ethnic cultures may maintain a positive relationship between their ethnic and national identification through positive intergroup orientations.

### Theoretical and practical implications

This study advances the literature on acculturation patterns [36] by presenting findings about the internal mechanism through which ethnic identity affects national identity. Theoretically, this study contributes to the model of orthogonality between ethnic and national identity [7], thereby helping to refute the unidimensional model that allows either ethnic maintenance or assimilation only. It not only suggests that greater identification with either one’s ethnic or national culture does not require lesser identification with the other but also identifies intergroup orientation as a mediator that strengthens the association between ethnic and national identification.

Practically, our findings suggest that state integration policies can have a decisive impact on the development of minority youth’s ethnic and national identification. An inclusive environment in which minority cultures are recognized and valued may be critical for minority youth’s development of a secure ethnic identity and may further contribute to their development of national belonging by fostering open and positive attitudes toward other national subgroups. For instance, implementing culturally responsive pedagogy at schools, which emphasizes creating an inclusive environment where minority cultures are not only recognized but celebrated, is vital for fostering a secure ethnic identity among minority youth, which, in turn, supports their integration into the national fabric. In addition, multicultural curriculums that respect and celebrate diverse cultural backgrounds and inter-ethnic community projects like intercultural communication workshops and youth camps play crucial roles. These initiatives promote mutual understanding and respect among diverse groups, which can develop positive attitudes toward other national subgroups. As many multiethnic countries have given priority to enhancing the sense of national community and preventing ethnic segregation, our findings may have important applicability across diverse multicultural settings.

### Limitations and future research

This study had several limitations that warrant discussion. First, the use of a survey research design meant that the study variables were measured at a single point in time. This cross-sectional design poses inherent limitations, especially when testing mediation effects, as it lacks time precedence, which is crucial for establishing causal relationships. This absence of temporal sequencing may lead to biased estimates and misinterpretations of the relationships between variables. Given that social identity and attitudes toward other groups are dynamic and can evolve, future research could benefit from employing a longitudinal design. This would not only enrich the findings of this study but also offer a more nuanced understanding of the ethnic and national identification and intergroup orientations of minority youth by considering the temporal variations in these constructs. In addition, the homogeneity of parental background and SES limited the generalizability of our findings. Future research with more diverse samples in terms of SES could explore the impact of these variables on the studied relationships.

Second, the exploration of the relationships between ethnic identity, national identity, and intergroup orientation was based on correlational rather than experimental evidence, and the reliance on self-report surveys to collect quantitative data poses its own set of challenges. Participants may exhibit social desirability bias, particularly in collectivistic cultures like China, where conformity to social norms and values is highly regarded. Therefore, future research should consider the application of experimental designs and mixed methods research approaches and employ data triangulation to gain more robust insights into the relationships between the study variables while mitigating the risks of bias inherent to self-reporting in collectivistic contexts.

Third, although the study had a relatively large sample size, the participants were recruited solely from a Yi minority region. Due to historical circumstances [61], the Yi region has had positive cultural exchanges with other ethnic groups in China since the Ming Dynasty (1368–1644). Such an environment enabled a relatively positive discourse regarding the combination of ethnic and national identities and facilitated the local government’s implementation of multiculturalism policies. However, the findings derived from our sample data may not be generalizable to minority youth outside the Yi region, especially for those with a conflicted identity orientation [26]. The disparities in multicultural policy application and ethnic group treatment across different regions of China highlight the complexities and nuances inherent in the nation’s multicultural landscape [62]. Future studies could focus on exploring and comparing the experiences and perceptions of ethnic minorities in different regions of China, where the implementation of multiculturalism policies and the discourse surrounding ethnic and national identities vary.
